# Management of Globus Pharyngeus

**DOI:** 10.1155/2013/946780

**Published:** 2013-07-11

**Authors:** S. Kortequee, P. D. Karkos, H. Atkinson, N. Sethi, D. C. Sylvester, R. S. Harar, S. Sood, W. J. Issing

**Affiliations:** ^1^Department of Otolaryngology, Bradford Royal Infirmary, Bradford BD9 6RJ, UK; ^2^Department of Otolaryngology, Pinderfields Hospital, Wakefield WF1 4DG, UK; ^3^Department of Otolaryngology, The Freeman Hospital, Newcastle upon Tyne NE7 7DN, UK

## Abstract

Globus pharyngeus is a common ENT condition. This paper reviews the current evidence on globus and gives a rational guide to the management of patients with globus. The aetiology of globus is still unclear though most ENT surgeons believe that reflux whether acidic or not plays a significant role. Though proton pump inhibitors are used extensively in practice, there is little evidence to support their efficacy. Most patients with globus can be discharged after simple office investigations. The role of pepsin-induced laryngeal injury is an exciting concept that needs further study. Given the benign nature of globus pharyngeus, in most cases, reassurance rather than treatment or extensive investigation with rigid oesophagoscopy or contrast swallows is all that is needed. We need more research into the aetiology of globus.

## 1. Introduction

Globus pharyngeus, the sensation of something stuck in the throat, has been noted since the time of Hippocrates. Purcell first used the term globus hystericus in the early 18th century [1]. In 1968, Malcomson [2] suggested the term globus pharyngeus as a more accurate description since not all patients with globus were either hysterical or female. 

Typically, globus is relieved by ingestion of solids or liquids and tends to be worse on dry swallows. Globus may be associated with throat irritation, soreness, dryness, catarrh, or constant throat clearing. It forms a large part of ENT practice and may account for about 4% of referrals to our outpatient clinics [3]. The prevalence is much higher in the general population as most people may not present to hospital with it. A recent study by Ali and Wilson [4] found that up to 78% of patients presenting to non-ENT clinics had had globus-type symptoms.

## 2. Aetiology

Despite the high prevalence in the community, the aetiology of globus remains unclear and highly controversial. It is slowly being accepted that it may be multifactorial and that when it occurs in isolation it rarely hides any sinister pathology [5]. Most of the recent work has suggested several mechanisms in isolation or not uncommonly in combination are to blame for the manifestation of globus pharyngeus; these include psychological factors, gastro-esophageal reflux (GOR), pharyngeal dysmotility, hypertonic upper oesophageal sphincter (UOS), and local anatomic abnormalities [6–11].

### 2.1. Psychological Factors

As its earlier name, globus hystericus, suggests, there has been a long history of links between globus and psychological factors. It is the fourth most discriminating symptom of a somatisation disorder after vomiting, aphonia, and painful extremities [12]. As most of the globus patients are quite rightly referred to ENT surgeons rather than to psychiatrists, a psychogenic basis must always be borne in mind. Gale et al. [13] in a detailed medical and psychological examinations including assessment with the Minnesota Multiphasic Personality Inventory (MMPI) of 4240 US male veterans demonstrated a 6.4% incidence of globus. This globus group scored higher in nine out of ten of the MMPI clinical scales. They concluded that in men there is a significant link to depression and somatization disorder and as a result other related treatable psychopathology should be investigated.

Harris et al. [14] when comparing globus patients with other ENT patients (as a control group) found that globus patients had had more severe life events in the year and less confiding relationships than controls. Social stress may thus play a role in either initiating or maintaining globus.

### 2.2. Reflux

The link between GOR and globus has been a matter of controversy for over forty years. Chevalier et al. [6] looked at globus patients with and without typical GOR symptoms. They found that 66.6% of the nonreflux globus group and 80% of the GOR globus group had significant episodes of reflux (based on pH monitoring). In direct contrast, Chen et al. in a similar study found no evidence of reflux in globus patients based on ambulatory pH monitoring [7].

Reflux is, however, best detected by impedance. Anandasabapathy and Jaffin [15] using multichannel intraluminal impedance and pH monitoring (MII-pH) have suggested that globus may also be due to nonacid (NAR) reflux. As MII-pH can detect reflux episodes independent of acid changes, it is allegedly more accurate at picking up proximal reflux. This latter study found NAR and proximal reflux to be significant predictors of globus.

Based on porcine models, pepsin has been shown to increase the levels of laryngeal protective proteins and thus explain the NAR link. Even though low pH is needed to activate pepsin, its stability means that it may be activated intracellularly or when the larynx is later exposed to acid [16].

### 2.3. Pharyngeal and Upper Oesophageal Sphincter Function (UOS)

Hypertonicity of the UOS has been suggested as a cause of globus, but several studies have yielded conflicting results. This has largely been due to possible technical difficulties in assessing UOS pressure profiles. It has long been recognised that the UOS pressure profile is asymmetrical, especially when using multilumen catheters. Therefore, earlier studies that have not taken this into account must be viewed with caution. Also, oral movement during swallowing and compression from surrounding structures complicates pressure readings.

UOS pressure measurements obtained using circumferential transducers are regarded as being more reflective of true intraluminal pressure. Sun et al. [17] looked at twenty-four healthy volunteers and thirty-two patients with globus and found UOS pressure to be normal in most of the globus patients and could not suggest it as a possible aetiological factor. Interestingly they found that videofluoroscopic evidence of pharyngeal dysfunction especially laryngeal penetration had a strong association with globus.

Tokashiki et al. [18], however, showed that perfusion of HCl into the distal oesophagus was related to a sensation of globus associated with a rise in UOS pressure. This rise in pressure was independent of the detection of a rise in pH in the hypopharynx.

### 2.4. Local Mechanical Abnormalities

Recently there have been reports of very subtle changes in anatomy that when rectified have given relief of globus.

Agada et al. [9] published a small series of patients with globus having “abnormally” retroverted epiglottises. The definition of a retroverted epiglottis is if the tip touches the tongue base when the tongue is protruded.

Ulug and Ulubil [10] have presented a case of corniculate cartilage subluxation presenting with globus. Other postulated causes include Eagles syndrome (calcified stylohyoid ligament), impalpable thyroid nodules [11], cervical osteophytes, lingual tonsils, or prominent greater cornu of the hyoid.

Gastric inlet patches have also been aetiologically linked to globus [19, 20]. These are congenital islands of ectopic gastric mucosa found in the cervical oesophagus. With the incidence of gastric inlet patch being quite common (3.6%), it is hard to establish a causal relationship. Alagozlu et al. [21] have gone further to suggest that it is *H. pylori* infection of the inlet patch that causes altered cervical perception and hence globus. What is worrying about this is that these patches have been associated with both squamous cell carcinomas and adenocarcinomas of the upper oesophagus [19, 22].

More interestingly though Shiomi et al. [12] looked at the mucus in the epipharynx of patients with globus and compared it with that from healthy volunteers, they found that there were significantly increased concentrations of fucose and sialic acid (the main determinants of mucus viscosity) in the mucus of those with globus as compared to normal subjects.

Lastly, though there is no evidence to suggest this, some ENT surgeons believe that globus may “simply” be a local sensory abnormality just like tinnitus.

## 3. Investigation

As with all our patients, the key is in taking a proper history. Pointers that would suggest sinister underlying pathology would include dysphagia, aspiration, regurgitation, weight loss, voice change, and pain. The presence of overt symptoms of GOR should be noted.

The head and neck should be thoroughly examined. This should include transnasal fibre-optic laryngoscopy (FOL) or if available transnasal flexible laryngooesophagoscopy (TNO). Any further investigation should be based on the findings at history and examination.

### 3.1. Radiology

In ENT departments in the UK, contrast swallows are the most popular radiological investigations used to investigate globus, with some departments historically using them to screen patients for upper aerodigestive tract malignancy [23, 24]. They have been favoured because they are safe (compared to rigid endoscopy), quick, and believed to increase diagnostic yield. 

Unfortunately there is particular concern that this modality may miss a malignancy. One of the authors (RPH) retrospectively reviewed a series of 1275 patients that had barium swallows [24]. Six hundred and ninety-nine patients had globus and 451 of these patients had globus without sinister symptoms. In these patients, barium swallows did not show any sinister pathology. Another review of barium swallows by Hajioff and Lowe [25] looked at 2854 barium swallows from two centres, and of the 2011 patients that presented with globus, none had a worrying abnormality on barium swallow. Only one retrospective case series [26] has found an association between isolated globus and hypopharyngeal cancer. Two cases out of twenty-three cases were retrospectively found to have malignancies (a piriform fossa and postcricoid tumour). More recent and larger studies have failed to make a similar association.

In the light of the previously mentioned we do not recommend barium swallows routinely for globus. The diagnostic yield for malignancy is poor though it may reassure the patient [27].

### 3.2. Endoscopy

Direct visualisation of the upper digestive tract is another means of investigating globus. The main drawback of this is that flexible oesophagoscopy often requires sedation, while rigid endoscopy requires a general anaesthetic and carries a small but significant risk of perforation.

Lorenz et al. [28] carried out flexible endoscopies on patients that had been referred by ENT for further investigation of globus, and all of the patients had had a normal outpatient ENT examination and barium swallow. 62.7% of the patients were found to have pathology that could possibly have caused their globus though no sinister pathology was noted. Similarly, Nagano et al. [29] in their study found a 36.5% incidence of benign oesophageal pathology in patients with globus on flexible endoscopy, but again no malignancies were identified.

Takwoingi et al. [30] retrospectively reviewed 250 patients that had undergone rigid endoscopy for globus. The most common recorded anomalies were cricopharyngeal spasm (4.8%) and reflux (4.4%). No tumours were found, and they concluded that rigid endoscopy played a limited role in the investigation of globus. One patient had a perforation that was successfully treated conservatively.

The most recent major advance in endoscopy is transnasal oesophagoscopy (TNO). It combines the main advantages of both conventional flexible and rigid oesophagoscopy with none of the major disadvantages. It can be done with just topical anaesthesia and vasoconstriction. There is total examination of the upper digestive tract down to the stomach with the ability to take biopsies at the same time. It has been shown to be safe with a high patient satisfaction rate [31].

Though TNO is not yet routinely available in the UK, we think that it is the ideal investigation for those ENT surgeons who want a relatively safe, cheap, and quick way of visualising the upper digestive tract especially the hypopharynx and postcricoid regions. Where TNO is available, almost 90% patients with globus can be discharged after their first visit [32]. We eagerly await studies comparing the diagnostic yield of TNO to that of rigid oesophagoscopy.

### 3.3. Symptom Scores and Indices

Despite the controversies, a large number of UK ENT surgeons believe that reflux plays a role in globus. Many of us do not use any scores or indices in our assessment of patients with globus [33]. The reflux symptom index and the reflux finding score are not particularly valid diagnostic tools when used in globus patients [34]. The Glasgow Edinburgh Throat Score (GETS) has been validated for use in globus but is not widely used [4].

### 3.4. Impedance and pH Studies

Because of the benign nature of globus, we rarely ever ask for pH or impedance studies in our patients. They often require referrals to the gastroenterologists and rarely contribute to our management plan. They are used mainly as a research tool. However, this may change in the future.

## 4. Treatment

Where there is uncertainty about the aetiology there will be uncertainty about the management. If patients have overt signs or symptoms suggestive of reflux in addition to globus, we would treat them aggressively with a proton pump inhibitor (PPi) twice daily and a reflux suppressant for at least 4 months [35]. We do not routinely use H2 receptor antagonists. A study from the Cleveland Clinic using a regimen similar to ours has been found to be effective in controlling the symptoms of laryngopharyngeal reflux (LPR). Most of the ENT surgeons in the UK seem to be prescribing sub-optimal doses of PPis [33].

In cases where there is globus but with no evidence of GOR, there is little merit in treating them with PPis. Two recent meta-analyses of the role of PPis in reflux related laryngeal disease have shown little or no benefit over placebo [36, 37]. They both recommend that more studies are required to define the subgroup of patients that will benefit from PPis.

PPis are useful in controlling symptoms secondary to gastric inlet mucosa. Where this fails, then argon plasma ablation has been useful in controlling symptoms [20]. *H. pylori* eradication therapy should also be performed if there was evidence of infection.

Speech and language therapists may have a role to play in managing globus patients. A few trials have shown that globus symptom scores do improve after a course of speech therapy [38, 39]. What is not clear from these studies is whether there is a specific effect from speech therapy or if improvement is due to increased reassurance. Hypnotically Assisted Relaxation (HAR) therapy has also been reported in a recent case series [40] to improve globus sensation regardless of the cause. Manometric UOS readings in the patients showed no change before and after HAR.

In cases where there are anatomical anomalies, the trend seems to be excision of the offending local structure, most often some part of the cartilaginous framework of the larynx [9, 10]. There have been surprisingly no issues with aspiration or voice change following these procedures. These results have to be viewed with caution as the numbers are small with short follow-up intervals.

We must also remember to assess the whole patient and make referrals to the psychiatrists where it is indicated. Therefore in most cases of globus, if the history and examination of the patient suggest no sinister pathology, then reassurance is often enough. Rowley showed that at 7 years about 55% of patients were asymptomatic and none had developed an upper aerodigestive tract malignancy [5]. At the present, we do not recommend any further radiologic or endoscopic examination for the patient with isolated globus.

## 5. Conclusion

Globus is a clinical diagnosis and not a diagnosis of exclusion. A complete head and neck examination including fibreoptic laryngoscopy is more than adequate to confidently discharge the classic globus pharyngeus patients. The introduction of TNO in one stop globus clinics has meant that with appropriate training otolaryngologists can nowadays and in selected cases complete a thorough upper aerodigestive tract examination, thus avoiding the need for any other investigations such as barium swallows or oesophagoscopies under general anaesthesia. Overinvestigating these patients can often add unnecessary stress to a group of patients who already seem to have higher levels of depression, anxiety, and other somatic concerns. In fact the authors believe that both barium swallow and panendoscopy under GA are things of the past and should not form part of the standard globus assessment.

More research needs to be carried out into the aetiology, treatment, and long-term prognosis of persistent globus.

## References

[1] Purcell J (1707). *A Treatise of Vapours or Hysterick Fits*.

[2] Malcomson KG (1968). Globus vel pharynges (a reconnaissance of proximal vagalmodalities). *The Journal of Laryngology & Otology*.

[3] Moloy PJ, Charter R (1982). The globus symptom. Incidence, therapeutic response, and age and sex relationships. *Archives of Otolaryngology*.

[4] Ali KHM, Wilson JA (2007). What is the severity of globus sensation in individuals who have never sought health care for it?. *Journal of Laryngology and Otology*.

[5] Rowley H, O’Dwyer TP, Jones AS, Timon CI (1995). The natural history of globus pharyngeus. *Laryngoscope*.

[6] Chevalier JM, Brossard E, Monnier P (2003). Globus sensation and gastroesophageal reflux. *European Archives of Oto-Rhino-Laryngology*.

[7] Chen C, Tsai C, Chou AS, Chiou J (2007). Utility of ambulatory pH monitoring and videofluoroscopy for the evaluation of patients with globus pharyngeus. *Dysphagia*.

[8] Corso MJ, Pursnani KG, Mohiuddin MA (1998). Globus sensation is associated with hypertensive upper esophageal sphincter but not with gastroesophageal reflux. *Digestive Diseases & Sciences*.

[9] Agada FO, Coatesworth AP, Grace ARH (2007). Retroverted epiglottis presenting as a variant of globus pharyngeus. *Journal of Laryngology and Otology*.

[10] Ulug T, Ulubil SA (2003). An unusual cause of foreign-body sensation in the throat: corniculate cartilage subluxation. *American Journal of Otolaryngology*.

[11] Marshall JN, McGann G, Cook JA, Taub N (1996). A prospective controlled study of high-resolution thyroid ultrasound in patients with globus pharyngeus. *Clinical Otolaryngology and Allied Sciences*.

[12] Shiomi Y, Oda N, Shiomi Y, Hosoda S (2002). Hyperviscoelasticity of epipharyngeal mucus may induce globus pharyngis. *Annals of Otology, Rhinology and Laryngology*.

[13] Gale CR, Wilson JA, Deary IJ (2009). Globus sensation and psychopathology in men: the vietnam experience study. *Psychosomatic Medicine*.

[14] Harris MB, Deary IJ, Wilson JA (1996). Life events and difficulties in relation to the onset of globus pharyngis. *Journal of Psychosomatic Research*.

[15] Anandasabapathy S, Jaffin BW (2006). Multichannel intraluminal impedance in the evaluation of patients with persistent globus on proton pump inhibitor therapy. *Annals of Otology, Rhinology and Laryngology*.

[16] Johnston N, Dettmar PW, Bishwokarma B, Lively MO, Koufman JA (2007). Activity/stability of human pepsin: implications for reflux attributed laryngeal disease. *Laryngoscope*.

[17] Sun J, Xu B, Yuan Y, Xu J (2002). Study on the function of pharynx & upper esophageal sphincter in globus hystericus. *World Journal of Gastroenterology*.

[18] Tokashiki R, Funato N, Suzuki M (2010). Globus sensation and increased upper esophageal sphincter pressure with distal esophageal acid perfusion. *European Archives of Oto-Rhino-Laryngology*.

[19] Alaani A, Jassar P, Warfield AT, Gouldesbrough DR, Smith I (2007). Heterotopic gastric mucosa in the cervical oesophagus (inlet patch) and globus pharyngeus—an under-recognised association. *Journal of Laryngology and Otology*.

[20] Meining A, Bajbouj M, Preeg M (2006). Argon plasma ablation of gastric inlet patches in the cervical esophagus may alleviate globus sensation: a pilot trial. *Endoscopy*.

[21] Alagozlu H, Simsek Z, Unal S, Cindoruk M, Dumlu S, Dursun A (2010). Is there an association between Helicobacter pylori in the inlet patch and globus sensation?. *World Journal of Gastroenterology*.

[22] Satoh S, Nakashima T, Watanabe K (2007). Hypopharyngeal squamous cell carcinoma bordering ectopic gastric mucosa “inlet patch” of the cervical esophagus. *Auris Nasus Larynx*.

[23] Back GW, Leong P, Kumar R, Corbridge R (2000). Value of barium swallow in investigation of globus pharyngeus. *Journal of Laryngology and Otology*.

[24] Harar RPS, Kumar S, Saeed MA, Gatland DJ (2004). Management of globus pharyngeus: review of 699 cases. *Journal of Laryngology and Otology*.

[25] Hajioff D, Lowe D (2004). The diagnostic value of barium swallow in globus syndrome. *International Journal of Clinical Practice*.

[26] Tsikoudas A, Ghuman N, Riad MA (2007). Globus sensation as early presentation of hypopharyngeal cancer. *Clinical Otolaryngology*.

[27] Mahrous AK, Kaoutzanis C, Amin K, Gluckman P (2012). Positive findings on barium swallow in patients presenting with a “sensation of a lump in the throat”. *European Archives of Oto-Rhino-Laryngology*.

[28] Lorenz R, Jorysz G, Clasen M (1993). The globus syndrome: value of flexible endoscopy of the upper gastrointestinal tract. *Journal of Laryngology and Otology*.

[29] Nagano H, Yoshifuku K, Kurono Y (2010). Association of a globus sensation with esophageal diseases. *Auris Nasus Larynx*.

[30] Takwoingi YM, Kale US, Morgan DW (2006). Rigid endoscopy in globus pharyngeus: how valuable is it?. *Journal of Laryngology and Otology*.

[31] Amin MR, Postma GN, Setzen M, Koufman JA (2008). Transnasal esophagoscopy: a position statement from the American Bronchoesophagological Association (ABEA). *Otolaryngology—Head and Neck Surgery*.

[32] Postma GN, Cohen JT, Belafsky PC (2005). Transnasal esophagoscopy: revisited (over 700 consecutive cases). *Laryngoscope*.

[33] Karkos PD, Benton J, Leong SC (2007). Trends in laryngopharyngeal reflux: a British ENT survey. *European Archives of Oto-Rhino-Laryngology*.

[34] Park KH, Choi SM, Kwon SUK, Yoon SW, Kim SUK (2006). Diagnosis of laryngopharyngeal reflux among globus patients. *Otolaryngology—Head and Neck Surgery*.

[35] McGlashan JA, Johnstone LM, Sykes J, Strugala V, Dettmar PW (2009). The value of a liquid alginate suspension (Gaviscon Advance) in the management of laryngopharyngeal reflux. *European Archives of Oto-Rhino-Laryngology*.

[36] Qadeer MA, Phillips CO, Lopez AR (2006). Proton pump inhibitor therapy for suspected GERD-related chronic laryngitis: a meta-analysis of randomized controlled trials. *American Journal of Gastroenterology*.

[37] Gatta L, Vaira D, Sorrenti G, Zucchini S, Sama C, Vakil N (2007). Meta-analysis: the efficacy of proton pump inhibitors for laryngeal symptoms attributed to gastro-oesophageal reflux disease. *Alimentary Pharmacology and Therapeutics*.

[38] Millichap F, Lee M, Pring T (2005). A lump in the throat: should speech and language therapists treat globus pharyngeus?. *Disability and Rehabilitation*.

[39] Khalil HS, Bridger MW, Hilton-Pierce M, Vincent J (2003). The use of speech therapy in the treatment of globus pharyngeus patients. A randomised controlled trial. *Revue de Laryngologie Otologie Rhinologie*.

[40] Kiebles JL, Kwiatek MA, Pandolfino JE, Kahrilas PJ, Keefer L (2010). Do patients with globus sensation respond to hypnotically assisted relaxation therapy? A case series report. *Diseases of the Esophagus*.

